# Overexpression of *Plasmodium falciparum* M1 Aminopeptidase Promotes an Increase in Intracellular Proteolysis and Modifies the Asexual Erythrocytic Cycle Development

**DOI:** 10.3390/pathogens10111452

**Published:** 2021-11-10

**Authors:** Carolina C. Hoff, Mauro F. Azevedo, Adriana B. Thurler, Sarah El Chamy Maluf, Pollyana M. S. Melo, Maday Alonso del Rivero, Jorge González-Bacerio, Adriana K. Carmona, Alexandre Budu, Marcos L. Gazarini

**Affiliations:** 1 Department of Biosciences, Federal University of São Paulo, Santos 11015-020, Brazil; caldascarol22@gmail.com (C.C.H.); maurousp@gmail.com (M.F.A.); 2 Department of Biophysics, Federal University of São Paulo, São Paulo 04039-032, Brazil; adrianathurler.epm81@gmail.com (A.B.T.); sarah.maluf08@gmail.com (S.E.C.M.); pollyana.saud@gmail.com (P.M.S.M.); ak.carmona@unifesp.br (A.K.C.); 3 Center for Protein Studies, Faculty of Biology, University of Havana, Vedado, La Habana 10400, Cuba; maday@fbio.uh.cu (M.A.d.R.); jogoba@fbio.uh.cu (J.G.-B.)

**Keywords:** *Plasmodium falciparum*, malaria, M1 alanyl-aminopeptidase, antimalarial, PfA-M1 overexpression

## Abstract

*Plasmodium falciparum*, the most virulent of the human malaria parasite, is responsible for high mortality rates worldwide. We studied the M1 alanyl-aminopeptidase of this protozoan (PfA-M1), which is involved in the final stages of hemoglobin cleavage, an essential process for parasite survival. Aiming to help in the rational development of drugs against this target, we developed a new strain of *P. falciparum* overexpressing PfA-M1 without the signal peptide (overPfA-M1). The overPfA-M1 parasites showed a 2.5-fold increase in proteolytic activity toward the fluorogenic substrate alanyl-7-amido-4-methylcoumarin, in relation to the wild-type group. Inhibition studies showed that overPfA-M1 presented a lower sensitivity against the metalloaminopeptidase inhibitor bestatin and to other recombinant PfA-M1 inhibitors, in comparison with the wild-type strain, indicating that PfA-M1 is a target for the in vitro antimalarial activity of these compounds. Moreover, overPfA-M1 parasites present a decreased in vitro growth, showing a reduced number of merozoites per schizont, and also a decrease in the iRBC area occupied by the parasite in trophozoite and schizont forms when compared to the controls. Interestingly, the transgenic parasite displays an increase in the aminopeptidase activity toward Met-, Ala-, Leu- and Arg-7-amido-4-methylcoumarin. We also investigated the potential role of calmodulin and cysteine proteases in PfA-M1 activity. Taken together, our data show that the overexpression of PfA-M1 in the parasite cytosol can be a suitable tool for the screening of antimalarials in specific high-throughput assays and may be used for the identification of intracellular molecular partners that modulate their activity in *P. falciparum*.

## 1. Introduction

Malaria is a disease caused by parasites of the genus *Plasmodium*, which affects millions of people worldwide, being responsible for hundreds of thousands of deaths each year [1]. Five protozoan species are known to infect humans (reviewed in [2]); however, the majority of malaria-related deaths are caused by *P. falciparum*. The major feature that contributes to the severity of the disease caused by these parasite species is the cytoadherence to the endothelial cells of capillaries, which in turn leads to the clogging of brain microvasculature (reviewed in [3]). Resistance to common drugs used to treat the disease, such as chloroquine and artemisinin combination therapy [4], highlight the importance of searching for new targets and antimalarial drugs.

The etiological agent of malaria is the female Anopheles mosquito. The infected female mosquito inoculates sporozoites in the dermis of the human host [5], which then reach the bloodstream and infect liver cells. After dividing, the resulting liver merozoites inside a merosome enter the bloodstream and infect erythrocytes inside which they undergo differentiation into ring, trophozoite, and schizont forms. The resulting merozoites burst the red blood cell and reinvade new erythrocytes, repeating the intraerythrocytic cycle (reviewed in [3]). The *P. falciparum* intraerythrocytic cycle spans 48 h. 

Peptidases play a pivotal role in parasite development, participating in the rupture of the erythrocyte membrane, in the invasion of red blood cells, and also in hemoglobin digestion [6,7]. Specifically, serine-proteases from the SERA family and subtilisins participate in egress and invasion of the host cell [7,8], while the hydrolysis of hemoglobin involves various classes of proteases: plasmepsins (aspartyl proteases), falcipains (cysteine proteases), falcilysins (metalloproteases), and aminopeptidases (serine and metalloproteases) [9,10,11,12]. Some proteases related to hemoglobin digestion are found inside the digestive vacuole, which possesses H^+^ ion pumps, maintaining an acidic pH [13], which is appropriate for the activity of these hydrolases. The digestive vacuole was identified as a calcium ion store [14,15]. Calcium is a ubiquitous second messenger (reviewed in [16]) that controls key events in the parasite life cycle, such as protein secretion, host cell invasion and egress (reviewed in [17]). The mitochondrion [18], the parasitophorous vacuole [19], and the endoplasmic reticulum [20] also participate in the homeostasis of this important second messenger. An important downstream effect of calcium signaling in *Plasmodium* is the modulation of proteolytic activity related to cysteine proteases [21,22,23,24]. Although the *Plasmodium* aminopeptidases occur in cell compartments with calcium fluctuations, a direct correlation with these enzymes’ activity is uncertain.

Four of the *P. falciparum* aminopeptidases are identified as methionyl-aminopeptidases (PFE1360c, PF10_0150, MAL8P1.140, PF14_0327), and could have a role in the hydrolysis of methionine from newly-synthesized polypeptides [25]. The remaining five enzymes are a post-prolylaminopeptidase (PF14_0015), a prolyl-aminopeptidase (PF14_0517), a leucyl-aminopeptidase (PF3D7_1446200), an aspartyl-aminopeptidase (PF3D7_0932300) and an alanyl-aminopeptidase (PF3D7_1311800). These aminopeptidases act in concert to hydrolyze hemoglobin [11].

In this work, we studied the M1 alanyl-aminopeptidase of *P. falciparum* (PfA-M1), an important and current antimalarial target. This protease, which belongs to the highly conserved M1 family of metalloproteases, is involved in the final stages of hemoglobin degradation and the gene knockout compromises the parasite development [11]. Some metalloaminopeptidase inhibitors have been tested in vitro and precluded parasite growth, highlighting the importance of these proteases as targets for the development of antimalarials [7,26,27,28,29] The cellular localization of PfA-M1 has been reported in different subcellular spaces such as cytosol, nucleus, and food vacuole [11,30,31,32]. As both calcium and aminopeptidases play crucial roles in *Plasmodium*, and calcium can be stored in the acidic compartment, where hemoglobin is hydrolyzed, we aimed to investigate the interplay between calcium and PfA-M1 in the *P. falciparum* intraerythrocytic cycle progression. Moreover, we intended to develop and characterize a parasite population overexpressing a cytosolic version of PfA-M1, which could be used for screening of drugs targeting this aminopeptidase.

## 2. Results

**Figure 1:**
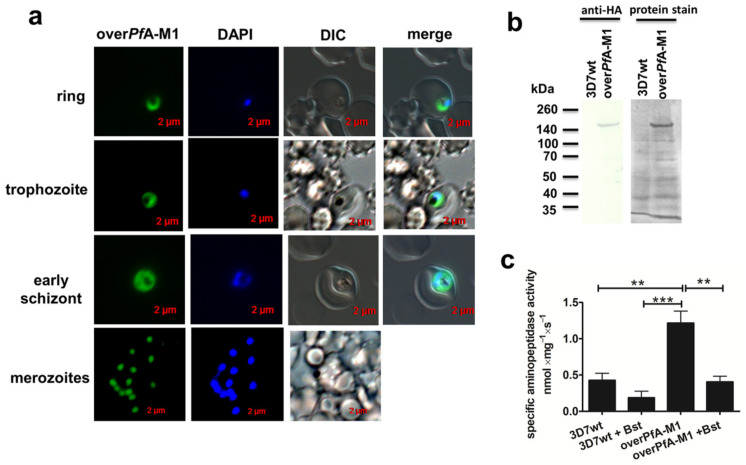
Assessment of overexpression of PfA-M1 in transgenic *P. falciparum* 3D7 parasite (overPfA-M1). (**a**) Green fluorescent protein (GFP) images of infected red blood cells overexpressing PfA-M1-GFP-HA (green). DNA was stained with DAPI (blue). (**b**) Western blot with an anti-HA antibody in overPfA-M1 and wild-type (3D7wt) parasites. (**c**) Specific aminopeptidase activity in 3D7wt and overPfA-M1 using Ala-AMC substrate and 10 μM of bestatin (Bst) was added at the last 2 min of measurement for slope stabilization. Data were compared with one-way ANOVA and Bonferroni *post test*; ** *p* < 0.001; *** *p* < 0.0001. Results are from three independent experiments.

A population of PfA-M1-GFP-HA-overexpressing *P. falciparum* 3D7 parasite (overPfA-M1; Figure 1) was obtained. The expression of PfA-M1-GFP-HA was confirmed via fluorescence microscopy (Figure 1a) and by western blot, using an anti-HA antibody (Figure 1b). Figure 1b shows a protein band of approximately 140 kDa that closely matches the predicted molecular mass for the fusion chimerical protein (135 kDa). OverPfA-M1 has a localization compatible with the cytosol, showing a homogeneous fluorescence distribution in the entire parasite cell and excluded from the hemozoin region (Figure 1a).

To confirm whether an increase in PfA-M1 activity resulted as a consequence of the overexpression of the aminopeptidase (i.e., that the overexpressed enzyme is catalytically active), we analyzed the cleavage of the PfA-M1-specific substrate Ala-AMC [33] by the transgenic population using the wild type (3D7wt) strain as a control, in synchronized trophozoites (Figure 1c). Indeed, the overPfA-M1 population displayed significantly higher proteolytic activity toward Ala-AMC (approximately 2.5 times higher), when compared to the 3D7wt strain (Figure 1c). Importantly, the catalytic activity was inhibited approximately 60% by bestatin, a specific inhibitor of metalloaminopeptidases (Figure 1c).

**Figure 2:**
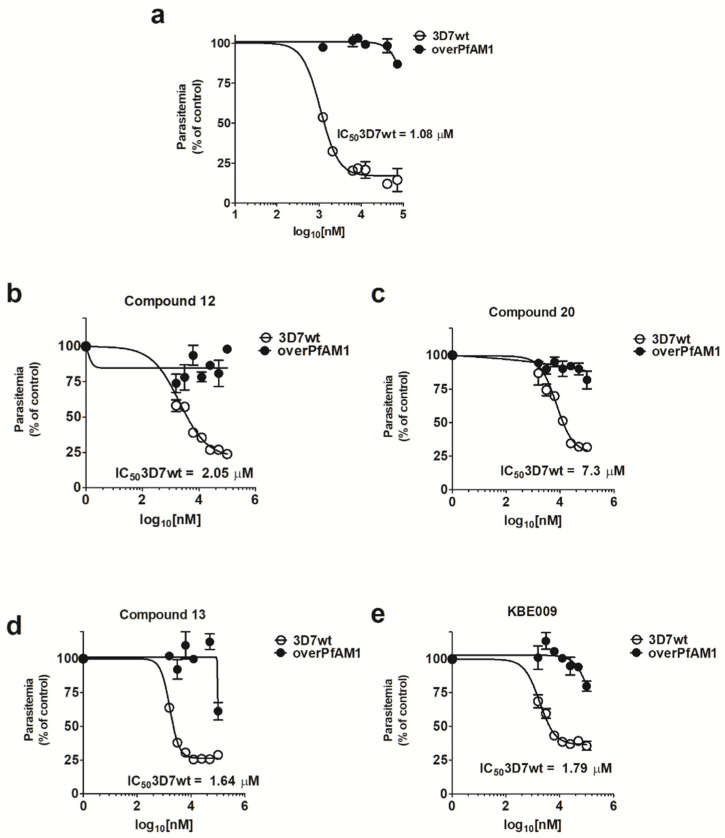
In vitro antimalarial effect of bestatin (**a**) and four recombinant PfA-M1 inhibitors (**b**–**e**) over transgenic (overPfA-M1) and wild-type (3D7wt) *P. falciparum* 3D7 parasites. Culture samples were quantified by FACS with bestatin at various concentrations, or in the presence of four different compounds. The parasite cultures were synchronized at the ring stage and adjusted to 0.5% hematocrit. Results are from three independent experiments.

Analysis of overPfA-M1 parasitemia demonstrated an increase in the bestatin IC50, in comparison with 3D7wt (Figure 2a). OverPfA-M1 parasites were resistant to bestatin concentrations higher than 100 μM, whereas this compound inhibited the 3D7wt growth with an IC50 of 1.08 μM. In addition, the transgenic parasite was more resistant to the antimalarial effect of the recombinant PfA-M1 inhibitors and in vitro antimalarial compounds 12, 20, 13, and KBE009 ([28]; Figure 2b–e).

**Figure 3:**
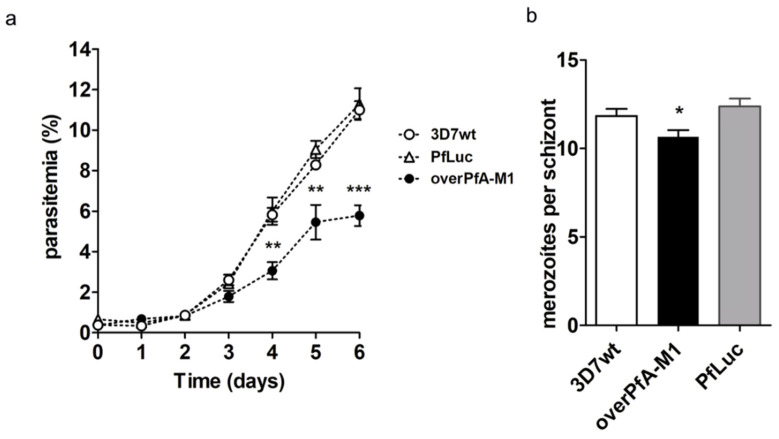
Growth of transgenic (overPfA-M1) *P. falciparum* 3D7 in comparison with wild-type (3D7wt) and luciferase overexpressing (PfLuc) parasites. Culture parasitemia evaluation every 24 h during 6 days (**a**). Parasites synchronized at the ring stage, with 0.5% initial parasitemia, were grown in the absence (for 3D7wt) or, presence of WR99210 (for PfLuc, overPfA-M1). Statistical analysis was performed through Fisher’s exact test. ** *p* < 0.001 and *** *p* < 0.0001 (*n* = 100 cells). Results are from three independent experiments. (**b**) Quantification of merozoites per schizont in infected erythrocytes for transgenic overexpressing PfA-M1 (overPfA-M1), luciferase (PfLuc) and wild-type (3D7wt) *P. falciparum* 3D7 parasite. Asynchronic cultures were maintained at 0.5% hematocrit and with parasitemia between 2 and 4%. Giemsa-stained smears were used to obtain 100 images of schizonts from each strain. Data from three independent experiments were compared with one-way ANOVA; * *p* < 0.01.

**Figure 4:**
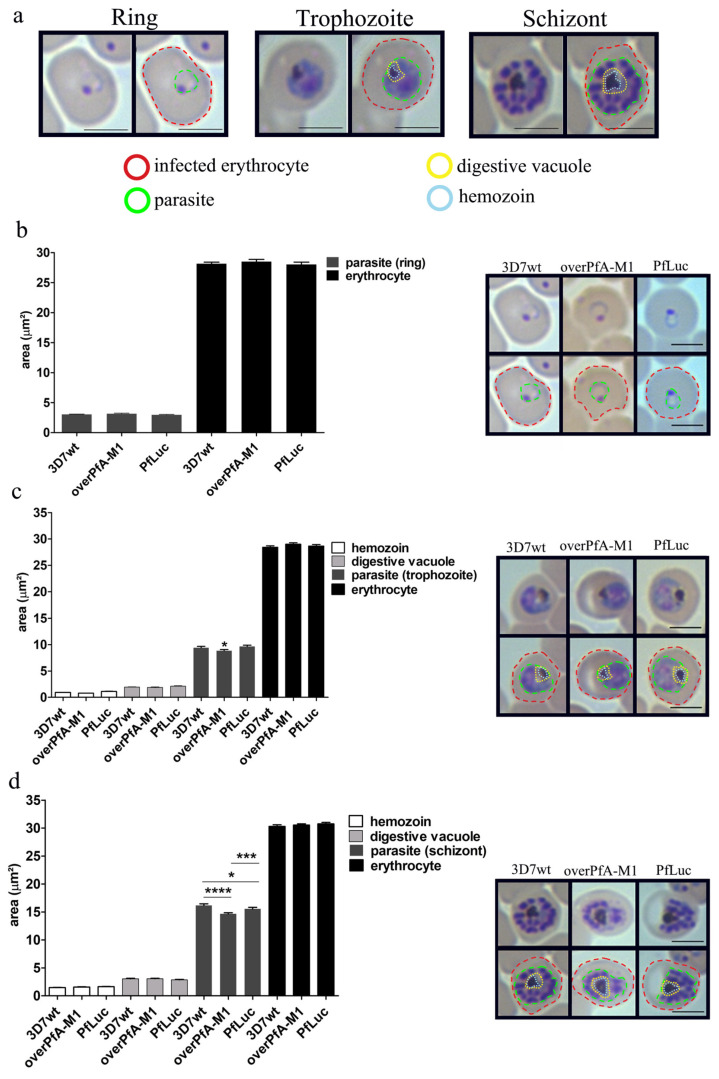
Evaluation of infected *Plasmodium falciparum* erythrocytes morphology. Measurement of hemozoin, digestive vacuole, parasite, and erythrocyte areas selected with different colored traces (**a**) rings (**b**), trophozoites (**c**), and schizonts (**d**) of transgenic overexpressing PfA-M1 (overPfA-M1), luciferase (PfLuc), and wild-type (3D7wt) *P. falciparum* 3D7 parasites. Asynchronous cultures were maintained at 0.5% hematocrit and 2–4% parasitemia. A representative image for each stage is shown. Data were analyzed by one-way ANOVA. * *p* < 0.05, *** *p* < 0.001 and **** *p* < 0.0001. Scale bar for all figures: 5 μm. The data shown were representative of *n* = 100 cells/experiment, from three independent experiments.

**Figure 5:**
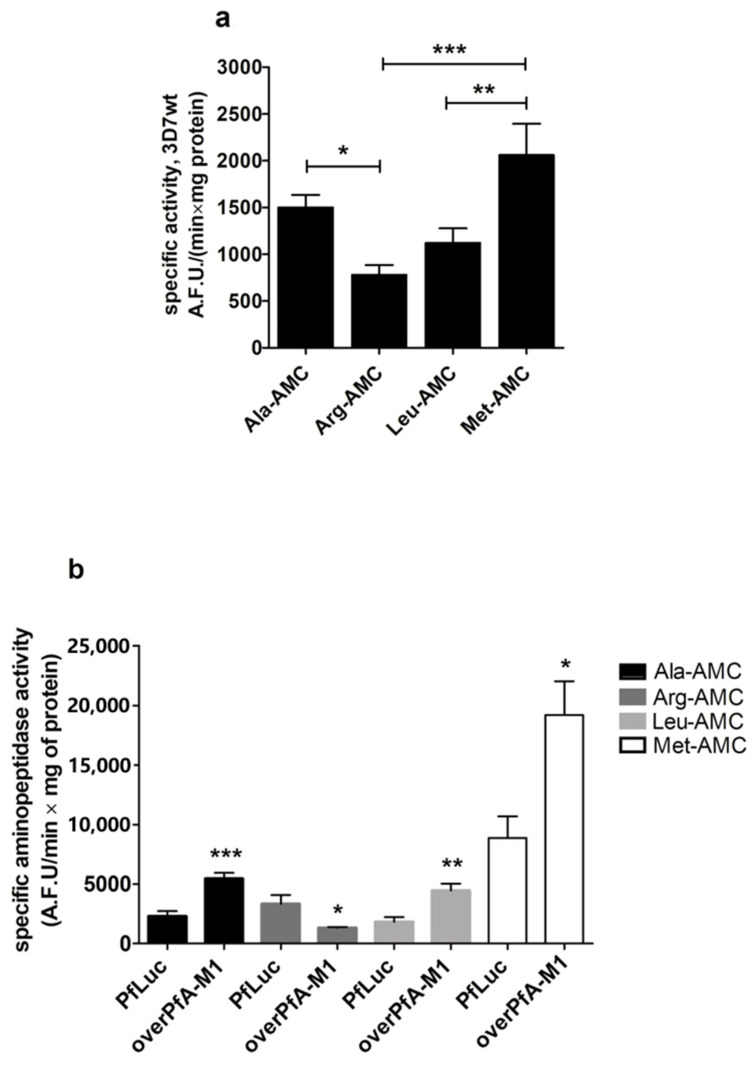
Specific intracellular aminopeptidase activity with various substrates in transgenic overexpressing PfA-M1 (overPfA-M1), luciferase (PfLuc), and wild-type (3D7wt) *P. falciparum* 3D7 parasites. Isolated trophozoite suspensions in buffer A were incubated with 10 μM of the fluorogenic substrates Ala-AMC, Arg-AMC, Leu-AMC or Met-AMC. (**a**) 3D7wt strain; (**b**) overPfA-M1 and PfLuc. * *p* < 0.05, ** *p* < 0.005 and *** *p* < 0.0007. Three independent experiments were performed.

The growth of overPfA-M1 in culture was slower when compared to either 3D7wt or a control strain, overexpressing luciferase fused to GFP-HA (PfLuc, Figure 3a). A significant decrease in the number of merozoites per schizont was also identified in the overPfA-M1 strain, in relation to both 3D7wt and PfLuc (Figure 3b). No significant decrease in the hemozoin or food vacuole areas were identified in the overPfA-M1, in comparison to the control strains. However, was observed a significant decrease in the size of trophozoites and schizonts in overPfA-M1, in relation to PfLuc and 3D7wt strains(Figure 4). The proteolytic activity in isolated trophozoites was assessed in the three strains, by using the fluorogenic substrates Ala-AMC, Arg-AMC, Leu-AMC, or Met-AMC. The substrate preference was Met-AMC > Ala-AMC > Leu-AMC > Arg-AMC in all strains (Figure 5a). However, the activity with Met-AMC was approximately 9-fold higher in overPfA-M1, compared to 3D7wt strain and 2-fold in relation to the PfLuc strain. The difference in proteolytic profiles between the luciferase and 3D7wt strains could be explained by off-target effects arising from transfection [34]. For Ala-AMC, the activity was 2.5-fold higher in the overPfA-M1 compared to the PfLuc strain (Figure 5b).

**Figure 6:**
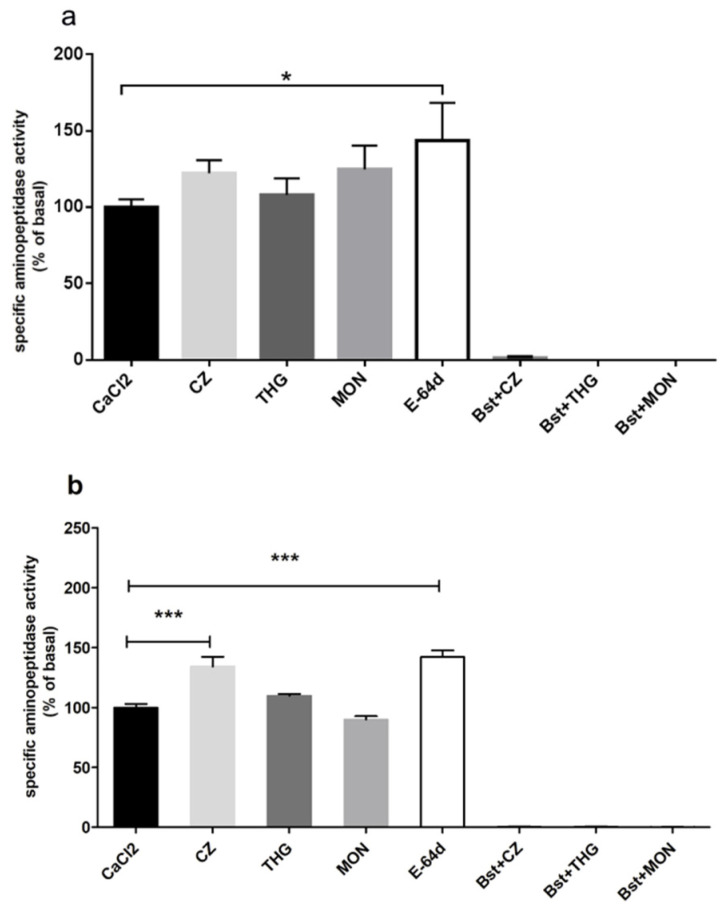
Proteolytic intracellular activity in *P. falciparum* 3D7(wt) parasites at the trophozoite stage with Ala-AMC (**a**) or Met-AMC (**b**) substrates. Synchronized parasites were isolated and incubated first with 50 μM bestatin for 15 min, and after with 10 μM calmidazolium (CZ), 10 μM thapsigargin (THG), 5 μM monensin (MON) or 10 μM E-64d for 10 min in buffer A supplemented with 5 mM CaCl_2_. 10 μM Ala-AMC or Met-AMC substrates were then added. Data were analyzed with one-way ANOVA. * *p* < 0.01; *** *p* < 0.0001. Data are from three independent experiments.

After the characterization of the aminopeptidase activity in the strains, we aimed to correlate it to the calcium homeostasis in the parasite. The calcium mobilization in the isolated, synchronized trophozoites was induced from intracellular calcium stores by employing thapsigargin, calmidazolium, or monensin. Neither of these inhibitors has altered the aminopeptidase activity, in relation to the basal levels of 3D7wt with the Ala-AMC substrate (Figure 6a). Interestingly, the cysteine protease inhibitor E64d, evoked an activity increase. Using the Met-AMC substrate, however, we observed that calmidazolium and E64d were able to elicit a ~35–40% increase in proteolysis (Figure 6b). Bestatin completely inhibited the proteolytic activity for both substrates (Figure 6).

## 3. Discussion

PfA-M1 is important for the intraerythrocytic development of *P. falciparum* and is a promising drug target [11,35]. Although it was not described as essential in a transposon mutagenesis screen [36], previous attempts of knockout of this gene have been unsuccessful, indicating an essential role of this aminopeptidase for parasite development [11]. We were able to overexpress in the parasite cytosol a functional N-terminal truncated version of PfA-M1 (i.e., without the 194 amino acids N-terminal extension and devoid of the signal peptide [30]) (Figure 1).

Dalal and Klemba [11] overexpressed PfA-M1 fused to the yellow fluorescent protein (YFP) in *P. falciparum* 3D7 by homologous recombination with a transfected episome. Transcription of this chimera’s gene was controlled by the endogenous PfA-M1 promoter, and a physiological pattern of expression was expected. In this context, the overexpressed aminopeptidase was localized in the food vacuole and the nucleus [11] as reported to the untagged PfA-M1, evaluated by immunofluorescence and cryo-immunoelectron microscopy using polyclonal anti-PfA-M1 antibodies [31]. The digestive vacuole localization can be explained by the expression of intact fusion protein PfA-M1-YFP (152 kDa) in parasites [11] since the N-terminal extension apparently contains a food vacuole localization signal [31]. In contrast, and in agreement with our results, a truncated PfA-M1 form (without the N-terminal extension and the food vacuole localization signal) fused to the antigenic epitope cmycB is actively overexpressed in *P. falciparum* D10 parasites as a 115 kDa product [37].

Since PfA-M1 is the main aminopeptidase in *P. falciparum* with activity against Ala-AMC [33], it increased activity in this substrate exhibited by overPfA-M1 parasite, compared to 3D7wt strongly indicates that the overexpressed enzyme is active (Figure 1c). In addition, the inhibition of this activity by bestatin (Figure 1c) supports this conclusion, since only PfA-M1 and PfA-M17 (the other neutral metalloaminopeptidase in *P. falciparum*) are bestatin-sensitive enzymes in the parasite [35], and PfA-M17 has a negligible activity against Ala-AMC [38].

Gardiner et al. did not demonstrate an increase in aminopeptidase activity in transgenic PfA-M1-overexpressing parasites or even a different sensitivity to bestatin compared with wild-type cells [39]. Although a protein of expected molecular mass (~120 kDa) was expressed, as confirmed by immunoblotting, it may have not been correctly folded and/or post-translationally modified to generate a functionally active enzyme. On the other hand, since the antimalarial compounds, such as bestatin, and compounds 12, 13, 20 and KBE009 inhibit recombinant PfA-M1 [28] and the increased resistance to these antimalarials exhibited by overPfA-M1, as shown in Figure 2, indicates that: (1) endogenous PfA-M1 is a target for the antimalarial activity of these compounds, and (2) PfA-M1 was overexpressed in a functional manner. Previously published results [40] are consistent with the presented data since increased PfA-M1 expression in the parasite cytosol protected *P. falciparum* from the growth inhibition caused by bestatin and compound 4 (another potent PfA-M1 inhibitor,). However, we cannot exclude the possibility that PfA-M1 overexpression diminishes the parasite sensitivity to bestatin and other PfA-M1 inhibitors by sequestering these compounds and preventing PfA-M17 inhibition. PfA-M17 is also a validated target in malaria and is inhibited by most PfA-M1 inhibitors [11,35].

The IC50 values of bestatin and the other recombinant PfA-M1 inhibitors obtained for the in vitro growth inhibition assay for 3D7wt strain (Figure 2) possesss some disparity from the reported by González-Bacerio et al. (IC50 for bestatin, 21 μM; KBE009, 18 μM; compound 20, 25 μM; compound 13, 26 μM; compound 12, 37 μM [28]). These differences are probably related to the medium supplementation used, namely Albumax I in the present study and human serum in case of González-Bacerio et al. work [28]. Differences in antimalarial compound IC50 values have also been reported by other authors comparing serum with non-serum substitutes in *Plasmodium* cultures [38]. These discrepancies could be due partially, to the disparity in lipid and protein contents between both culture media, and the respective differential properties of antimalarials binding to them, which in turn can influence the drug pharmacodynamic profile. An illustrative example is halofantrine, a highly lipophylic drug that significantly associates with the triacylglyceride-rich plasmatic lipoproteins [41]. According to this, bestatin and the other PfA-M1 inhibitors could interact with some serum components, reducing the effective concentration of the compounds and increasing the IC50 in comparison with the Albumax I-supplemented medium. Notwithstanding these differences, the IC50 obtained in this work allows establishing a comparison between both strains, because they were treated at the same conditions.

The overexpression of PfA-M1 led to the modification of the *P. falciparum* phenotype. It exhibits a lower parasite volume (Figure 4b,c) and merozoites number per schizont, in comparison with 3D7wt (Figure 4). This potentially compromises cell viability, which could account for the reduced parasite growth (Figure 3). It is also possible that the smaller parasite size is related to the slow growth. 

The role of PfA-M1 in hemoglobin hydrolysis is well known [11,31,35,42]. The enzyme overexpression may accelerate the hydrolysis of hemoglobin, generating a surge in metabolites and osmotic stress [43]. It is also possible that PfA-M1 could have a role in the nucleus [11,31], controlling mitosis, as observed for the aminopeptidase A in mammals [44], however, it needs to be confirmed for PfA-M1.

Poreba et al. [33] have shown that the purified recombinant PfA-M1 preferentially cleaves the N-terminal peptide bond of methionine, leucine, alanine, and arginine (leucine being also cleaved by PfA-M17). González-Bacerio et al. [45] have shown that the preference order for substrates was Met > Arg > Ala > Leu at neutral pH for another recombinant form of PfA-M1 while our study has shown a preference for Met > Ala > Leu > Arg for endogenous PfA-M1 in isolated trophozoites (Figure 5a). This difference could be explained by the diverse enzyme forms (recombinant vs. native) and the different experimental conditions in terms of pH, ionic strength, and so forth, in both assays (in vitro enzymatic assay vs. parasite intracellular environment).

We performed proteolysis experiments using Met-AMC and Ala-AMC, because these substrates suffered a higher rate of processing compared to the controls in our model (Figure 5). Monensin, a Ca^2+^/H^+^ ionophore that diminishes pH in the cytosol [46,47] did not affect proteolysis (Figure 6). This could be explained by the fact that parasites were maintained at a neutral pH buffer and the drug probably did not diminish the pH to an extent to affect the aminopeptidase activity. Thapsigargin, a SERCA inhibitor that leads to an increase of calcium in the cytosol [48] also had no effect upon aminopeptidase activity (Figure 6), suggesting that calcium does not directly control aminopeptidase activity against these substrates.

On the other hand, calmidazolium, a calmodulin inhibitor and an important calcium sensor in *Plasmodium* [24], was able to increase aminopeptidase activity when the Met-AMC substrate was used (Figure 6b). Interestingly, E64d, a cysteine protease inhibitor [49] also led to an increase in Ala-AMC and Met-AMC hydrolysis (Figure 6). These results suggest that the aminopeptidase activity in *P. falciparum* is altered by changes in the intracellular substrate pool after the treatment with cysteine proteases or calmodulin inhibitors, which is completely different for untreated parasites, allowing a favorable hydrolysis of non-native substrates, which were not normally available to PfA-M1.

## 4. Materials and Methods

### 4.1. P. falciparum Culture and Synchronization

Erythrocytic stages of *P. falciparum* 3D7 were maintained in a culture based on a method previously described by Trager and Jensen [50]. Briefly, parasites were grown in RPMI 1640 medium (10.4 g/L; Gibco, Waltham, MA, USA) containing 0.25% (*m*/*v*) sodium bicarbonate (pH 7.4; Gibco, USA), supplemented with Albumax I (Gibco, Auckland, New Zealand), under a controlled atmosphere of 3% O_2_, 5% CO_2_ and 92% N_2_, at 37 °C using an incubator (Thermo Electron Corporation, Hepa Class 100, Marietta, OH, USA). Daily changes of the culture medium were performed. Fresh human erythrocytes (less than one-month-old), obtained from healthy adult donors using standard protocols, were used as host cells at 0.5% hematocrit. Parasite viability, growth stage, and parasitemia level were monitored by microscopic observation of the cell smears fixed with methanol and stained with Giemsa dye.

When necessary, the synchronization of parasites was obtained by the sorbitol method based on Lambros and Vanderberg (1979) [51]. The parasite-infected erythrocytes were centrifuged (500× *g*, 5 min), the media removed and the cells resuspended in 10 volumes of 37 °C-pre-warmed D-sorbitol (5%, *m*/*v*) solution for 5 min at room temperature in shaking (240 rpm). The culture was then centrifuged, sorbitol was removed and the infected erythrocytes were cultured as previously described. Synchronization was verified by microscopic observation of the cell smears fixed with methanol and stained with Giemsa dye. As a result of the application of this protocol, cultures with 88–90% parasites at the ring stage were obtained. The synchronized parasites were maintained for 48 h before performing the next experiment.

### 4.2. Cloning of PfA-M1 Gene for Overexpression in P. falciparum

The PfA-M1 gene sequence corresponding to the residues 195–1085, without the signal peptide, codon-optimized for *E. coli* expression [45], was amplified via PCR using restriction sites for Xho I, underlined (CTCGAGATGGAACCGAAAATTCATTATCGCA) and Pst I, underlined (CTGCAGCCAGTTTATTGGTCAGGCGC). The stop codon was removed in order to allow 3′ fusion of the protein with the green fluorescent protein (GFP) and hemagglutinin (HA). The PfA-M1 was cloned in pcr 2.1 vector (TOPO-TA system, Life Technologies, Carlsbad, CA, USA) according to the manufacturer’s protocol. The open reading frame was then subcloned in the pEF-GFP vector [52] in 3′ fusion with GFP and HA and under the control of the calmodulin promoter, allowing overexpression of PfA-M1 in the intraerythrocytic cycle of *P. falciparum* (Supplemental File S1).

### 4.3. Transfection and Selection of PfA-M1-Overexpressing P. falciparum

The transfection of the pEF-PfA-M1-GFP-HA in *P. falciparum* (3D7 strain) was performed according to a protocol modified from Fidock and Wellems [53]. The selection of parasites containing the plasmid was obtained by their maintenance in the presence of 10 nM WR99210 [53].

### 4.4. Western Blot and Microscopic Detection of overPfA-M1 Parasites

The transgenic overPfA-M1-overexpressing parasites were washed with PBS (137 mM NaCl, 2.7 mM KCl, 4.3 mM Na_2_HPO_4_, 1.4 mM NaH_2_PO_4_) and stained for 10 min with DAPI (5 μg/mL). Images were acquired with a fluorescence microscope (AxioObserver Z1, Carl Zeiss, 63× objective, Jena, Germany), using DAPI and GFP filters.

Western blot was performed with parasite protein extracts. First, parasites were isolated from erythrocytes with saponin. The isolation was performed from 100 mL cultures at 5% parasitemia and 0.5% hematocrit. The infected erythrocytes were washed twice with ice-cold PBS, centrifuging 10 min at 300× *g*, and selectively lysed using 0.01% (*w/v*) saponin (Sigma, St Louis, MO, USA) in ice-cold PBS. The resultant suspension was centrifuged at 3300× *g* for 10 min at 4 °C. The parasite pellet was washed twice, resuspended in 1 mL ice-cold PBS, and kept on ice. Morphology of the isolated parasites was verified by microscopic observation of the cell smears fixed with methanol and stained with Giemsa dye.

Next, parasites were lysed in the BugBuster (Merck, Darmstadt, Germany) buffer, supplemented with protease inhibitors. The samples were incubated on ice for 30 min and centrifuged at 10,000× *g*, for 10 min. The protein from the supernatant was quantified through the Bradford method [54]. Twenty-five μg of protein was denatured through heating at 95 °C, for 10 min in Laemmli buffer and submitted to SDS-PAGE (10%). After transfer to a PVDF membrane and blocking overnight at 4 °C with PBS containing 0.05% Tween-20 (*v*/*v*, PBS-T) and 5% (*w/v*) bovine serum albumin (BSA, Sigma, St Louis, MO, USA), the membrane was incubated for 2 h at room temperature in PBS-T containing 5% (*w*/*v*) BSA (Sigma) and 1:2000 anti-HA (Sigma). After washing, the membrane was incubated with anti-IgG rabbit-HRP (Sigma) 1:1000, in PBS-T, for 1 h at room temperature. After three washes in the same buffer, the membrane was exposed to TMB substrate (KPL, Rockford, IL, USA). The loading control of the membrane was performed by staining with the MemCode kit (Thermo Scientific, Waltham, MA, USA).

### 4.5. Assessment of overPfA-M1 Aminopeptidase Activity, Inhibition by Bestatin and Relationship with Ca^2+^ Mobilizers and E64d in Isolated Live Parasites

In order to detect the hydrolytic activity of overPfA-M1 in the cultures, wild-type and transgenic overPfA-M1-overexpressing parasites were isolated with saponin as previously described and were incubated with 10 μM of the fluorogenic substrate Ala-7-amido-4-methylcoumarin (AMC; ≈0.05 K_M_ for native PfA-M1; [42]) in buffer A (116 mM NaCl, 5.4 mM KCl, 0.8 mM MgSO_4_, 50 mM MOPS, 5 mM CaCl_2_, 5.5 mM D-glucose pH 7.4), not more than 3 h after the parasite isolation. The proteolytic activity was measured in a 1 mL cuvette, at 37 °C, in a fluorimeter (Hitachi F7000, Tokyo, Japan) for 10 min, under agitation, by measuring the fluorescence at λex 380 nm and λem 460 nm. The enzyme activity present in the volume of parasite suspension (10 µL) without inhibitors or compounds is linearly related to the initial rate. Only the linear portions of the progress curves, corresponding to a substrate consumption lower than 5%, were used to measure the reaction rates. The slopes with R^2^ < 0.98 were not considered.

At approximately the last 2 min of measurement, 10 μM of the metalloaminopeptidase inhibitor, bestatin was added to assess the inhibition slope. The substrate and inhibitor were solubilized in DMSO, and the solvent final concentration was not higher than 2% (*v*/*v*). Fluorescence was converted into μM of product based on a calibration curve obtained from complete hydrolysis of 1, 2, 5, and 10 μM Ala-AMC, subtracting the background values corresponding to the non-hydrolyzed substrate controls. Non-infected erythrocytes treated in the same manner were used as a negative control for activity. Each experiment was performed in triplicate.

Saponin-isolated wild-type and transgenic PfA-M1- and luciferase-overexpressing parasites were analyzed at the trophozoite stage (10^7^ cells/mL, resuspended in buffer A, 200 μL/well in black 96-well ELISA plates). First, aminopeptidase activity was measured toward Ala-, Arg-, Met- or Leu-AMC substrates (AminoTech P & D, São Paulo, Brazil). In another experiment using only the wild-type strain, 50 μM bestatin was added to the parasites and incubated for 15 min in the presence of 5 mM CaCl_2_. After, 10 μM calmidazolium, a calmodulin inhibitor; 10 μM thapsigargin, a SERCA inhibitor; 5 μM monensin, a H^+^/Ca^2+^ ionophore; and 10 μM E-64d, a cysteine protease inhibitor, were added to the parasites and incubated for 10 min (E64d was added without previous incubation with bestatin). Then, 10 μM of the fluorogenic substrates Ala-AMC or Met-AMC were added. Enzymatic activity was measured as described above. Protein concentration was measured using the Bradford method [54] to assess the specific activity [45,55]. In the second experiment, basal activity was determined in the presence only of 5 mM CaCl_2_. These experiments were performed in triplicate.

### 4.6. In Vitro Antimalarial Activity Assays

The antimalarial activity assays were performed on 96-well ELISA plates (200 μL per well), using 2 μL bestatin or compounds 12, 13, 20, and KBE009 [28] solubilized in DMSO. Hence, the solvent final concentration was 1% (*v*/*v*). Synchronized cultures of *P. falciparum* 3D7 (wild-type and transgenic PfA-M1-overexpressing parasites), at the ring stage and 0.5% hematocrit, were incubated with bestatin or the compounds at various concentrations in the range 1–100 μM for 72 h, at 37 °C. The supernatant was removed and the cells were fixed with 2% (*v*/*v*) formaldehyde in PBS. After 24 h at room temperature, PBS containing 0.1% Triton X-100 and 1 nM YOYO-1 DNA probe was added. Analysis of parasitemia was performed in the FACSCalibur cytometer (BD, San Jose, CA, USA; excitation: 488 nm; 10,000 cells counted), following the protocol described in Schuck et al. [56].

Parasite growth was quantified using the Cyflogic software (version 1.2.1; CyFlo Ltd. http://www.cyflogic.com, accessed on 5 November 2021). Non-infected erythrocytes provided the background signal. Growth values were normalized using the growth of DMSO-treated parasites (without compound) as 100%. Non-treated infected red blood cells were used to confirm that 1% (*v*/*v*) DMSO does not affect the growth, by comparing with the DMSO controls. The IC50 values were calculated by the nonlinear fit of the dose-inhibition function to the experimental data, using GraphPad Prism 6 software (GraphPad Inc, San Diego, CA, USA). All assays were performed at least in triplicate.

### 4.7. Morphology Analysis of P. falciparum

After the staining of infected erythrocyte smears with Giemsa, morphometric analysis of the areas (μm^2^) of the infected erythrocyte and wild-type and transgenic overPfA-M1- and luciferase-overexpressing parasites in all stages (ring, trophozoite, and schizont) were measured in ZEN 2 software (Carl Zeiss). In the trophozoite and schizont stages, the digestive vacuole and hemozoin areas were also measured. One-hundred infected erythrocytes were measured in each strain and parasite stage. The images were acquired in a PrimoStar microscope (Zeiss), equipped with an Axiocam 105 color camera.

### 4.8. Analysis of Parasitemia in P. falciparum

Wild-type and transgenic PfA-M1 and luciferase-overexpressing parasites were synchronized to the ring stage and adjusted to 0.5% parasitemia and 0.5% hematocrit. The samples for analysis were collected every 24 h for 6 days. The samples were fixed in 2% formaldehyde (*v*/*v*) for cytometric evaluation in BD Facs Aria II, and were confirmed by counting in Giemsa-stained smears.

### 4.9. Statistical Analysis

For statistical analysis of the data, GraphPad Prism 6 (GraphPad Inc., San Diego, CA, USA) was employed. t-Student and one-way ANOVA followed by the Newman–Keuls post-test were used, as indicated. The statistical significance threshold was *p* = 0.05. Data are presented as mean ± S.E.M.

## 5. Conclusions

Our results suggest that the aminopeptidase activity against Ala-AMC and Met-AMC in *P. falciparum* are not directly modulated by Ca^+2^, and the enhancement of PfA-M1 activity resultant from calmodulin and cysteine protease inhibition could be due to the generation of an altered substrate pool previously not available to the aminopeptidase. PfA-M1 overexpression changes the *P. falciparum* phenotype in its erythrocytic stages (diminishes the in vitro growth speed, the size of trophozoites and schizonts, the number of merozoites per schizont and increase the aminopeptidase activity toward Met-, Ala-, Leu- and Arg-AMC), probably by augmenting either hemoglobin hydrolysis or osmotic stress. Of note, we developed a population of *P. falciparum* overexpressing active PfA-M1, which can be a suitable tool for the screening of potent antimalarials in specific high-throughput assays targeted to this essential aminopeptidase. Transgenic overexpressing parasites can also be used to confirm that endogenous PfA-M1 is a target for the in vitro antimalarial activity of recombinant PfA-M1 inhibitors.

## Supplementary Materials

The following are available online at https://www.mdpi.com/article/10.3390/pathogens10111452/s1, File S1: PEF-PfA-M1-HA GFP plasmid with highlighted features. PfA-M1 is devoid of its signal peptide-coding sequence and in frame with GFP and 3xHA at its C-terminal portion.
