# Supplementary material for: Overexpression of Plasmodium falciparum M1 Aminopeptidase Promotes an Increase in Intracellular Proteolysis and Modifies the Asexual Erythrocytic Cycle Development
Source: Pathogens. 2021 Nov 10;10(11):1452. doi: 10.3390/pathogens10111452 (PMC8618464; doi:10.3390/pathogens10111452)
Supplement: Supplementary file 1 [file pathogens-10-01452-s001.zip › pathogens-1397588-File S1.pdf]

PEF-PfA-M1-GPF plasmid sequence

CTAGCTTAATTCTTTCCGAGCTCTTTATGCTTAAGTTTACAATTTAATATTCATACTTTAAGTATTTTTTGT
AGTATCCTAGATATTGTGCTTTAAATGCTCACCCCTCAAAGCACCAGTAATATTTTCATCCACTGAAATA
CCATTAAATTTTCAAAAAAATACTATGCATATAATGTTATACATATAAACATAAAACGCCATGTAAATCA
AAAAATATATAAAAATATGTATAAAAATAAATATGCACTAAATATAAGCTAATTATGCATAAAAATTAA
AGTGCCCTTTATTAACTAGCTAGTCGTAATTATTTATATTTCTATGTTATAAAAAAATCCTCATATAATAA
TATAATTAATATATGTAATGTTTTTTTATTTTATAATTTTAATATAAAATAATATGTAAATTAATTCAAAA
AATAAATATAATTGTTGTGAAACAAAAAACGTAATTTTTTCATTTGCCTTCAAAATTTAAATTTATTTTAA
TATTTCCTAAAATATATATACTTTGTGTATAAATATATAAAAATATATATTTGCTTATAAATAAATAAAAA
TTTTATAAAACTCGAGATGGAACCGAAAATTCATTATCGCAAAGATTATAAACCGAGCGGCTTTATTATT
AATAATGTGACCCTGAACATTAATATTCATGATAATGAAACCATTGTGCGCAGCGTTCTGGATATGGAT
ATTTCAAAACATAATGTGGGCGAAGATCTGGTTTTTGATGGTGTTGGCCTGAAAATTAATGAAATTAGC
ATCAATAATAAAAAACTGGTGGAAGGCGAAGAATATACCTATGATAATGAATTTCTGACCATTTTTAGC
AAATTTGTGCCGAAAAGCAAATTTGCCTTTAGCAGCGAAGTGATTATTCATCCGGAAACCAATTATGCA
CTGACCGGTCTGTATAAAAGCAAAAATATTATTGTGAGCCAGTGCGAAGCAACCGGTTTTCGTCGTATT
ACCTTTTTTATTGATCGTCCGGATATGATGGCCAAATATGATGTTACCGTGACCGCAGATAAAGAAAAA
TATCCGGTTCTGCTGAGCAATGGCGATAAAGTTAATGAATTTGAAATTCCGGGTGGTCGTCATGGTGCA
CGTTTTAATGATCCGCATCTGAAACCGTGTTACCTGTTTGCAGTTGTTGCCGGTGATCTGAAACATCTGA
GCGCAACCTATATTACCAAATATACCAAAAAAAAAGTGGAACTGTATGTGTTTAGCGAAGAAAAATATG
TGAGCAAACTGCAATGGGCACTGGAATGTCTGAAAAAAAGCATGGCCTTTGATGAAGATTATTTTGGC
CTGGAATATGATCTGAGCCGTCTGAATCTGGTTGCAGTTAGCGATTTTAATGTTGGTGCCATGGAAAAT
AAAGGCCTGAATATTTTTAATGCCAATAGCCTGCTGGCCAGCAAAAAAAATAGCATTGATTTTAGCTAT
GCCCGTATTCTGACCGTTGTTGGTCATGAATACTTCCATAATTATACCGGCAATCGTGTGACCCTGCGTG
ATTGGTTTCAGCTGACCCTGAAAGAAGGTCTGACCGTTCATCGTGAAAACCTGTTTAGTGAAGAAATGA
CCAAAACCGTTACCACCCGTCTGAGCCATGTTGATCTGCTGCGTAGCGTTCAGTTTCTGGAAGATAGCA
GTCCGCTGAGCCATCCGATTCGTCCGGAAAGCTATGTTAGCATGGAAAATTTTTATACCACCACCGTGT
ATGATAAAGGTAGCGAAGTTATGCGTATGTATCTGACCATTCTGGGTGAAGAATACTACAAAAAAGGC
TTCGACATCTACATCAAAAAAAATGATGGCAATACCGCGACCTGCGAAGATTTTAATTATGCAATGGAA
CAGGCCTATAAAATGAAAAAAGCCGATAATAGCGCCAATCTGAATCAGTATCTGCTGTGGTTTAGCCAG
AGCGGTACACCGCATGTTAGCTTTAAATATAATTATGATGCCGAAAAAAAACAGTATAGCATTCATGTG
AATCAGTATACCAAACCGGATGAAAATCAGAAAGAAAAAAAACCGCTGTTTATTCCGATTAGCGTGGG
TCTGATTAATCCGGAAAATGGCAAAGAAATGATTAGCCAGACCACCCTGGAACTGACCAAAGAAAGCG
ATACCTTTGTGTTTAATAATATTGCCGTGAAACCGATTCCGAGCCTGTTTCGTGGTTTTAGCGCACCGGT
TTATATTGAAGATAATCTGACCGATGAAGAACGTATTCTGCTGCTGAAATATGATAGTGATGCCTTTGT
GCGCTATAATAGCTGCACCAATATTTATATGAAGCAAATCCTGATGAACTATAATGAATTTCTGAAAGC
CAAAAATGAAAAACTGGAATCCTTTAATCTGACACCGGTTAATGCCCAGTTTATTGATGCCATTAAATAT
CTGCTGGAAGATCCGCATGCAGATGCAGGTTTTAAAAGCTATATTGTTAGCCTGCCGCAGGATCGCTAT
ATTATTAATTTTGTGAGCAATCTGGATACCGATGTTCTGGCAGATACCAAAGAATATATTTATAAACAAA
TTGGCGATAAACTGAATGATGTGTATTATAAAATGTTTAAAAGCCTGGAAGCCAAAGCAGATGATCTG
ACCTATTTTAATGATGAAAGCCATGTGGATTTTGATCAGATGAATATGCGTACCCTGCGTAATACCCTGC
TGAGCCTGCTGTCAAAAGCACAGTATCCGAATATTCTGAATGAAATTATTGAACATAGCAAAAGCCCGT
ATCCGAGCAATTGGCTGACCAGCCTGAGCGTTAGCGCATACTTCGATAAATATTTCGAACTGTACGATA
AAACCTATAAACTGAGCAAAGATGATGAACTGCTGCTGCAAGAATGGCTGAAAACCGTTAGCCGTAGC

GATCGTAAAGATATTTATGAAATTCTGAAAAAACTGGAAAATGAAGTGCTGAAAGATAGCAAAAATCC
GAATGATATTCGTGCAGTGTATCTGCCGTTTACCAATAATCTGCGTCGCTTTCATGATATTAGCGGCAAA
GGCTATAAACTGATTGCCGAAGTTATTACCAAAACCGATAAATTTAATCCGATGGTTGCAACCCAGCTG
TGTGAACCGTTTAAACTGTGGAATAAACTGGATACCAAACGTCAAGAACTGATGCTGAATGAAATGAA
TACCATGCTGCAAGAACCGAATATTAGCAATAATCTGAAAGAATATCTGCTGCGCCTGACCAATAAACT
GGCTGCAGAAAAAATGGCTACACGTGCAAGTGCAGCTATGAGTAAAGGAGAAGAACTTTTCACTGGA
GTTGTCCCAATTCTTGTTGAATTAGATGGTGATGTTAATGGGCACAAATTTTCTGTCAGTGGAGAGGGT
GAAGGTGATGCAACATACGGAAAACTTACCCTTAAATTTATTTGCACTACTGGAAAACTACCTGTTCCAT
GGCCAACACTTGTCACTACTTTCGCGTATGGTCTTCAATGCTTTGCGAGATACCCAGATCATATGAAACA
GCATGACTTTTTCAAGAGTGCCATGCCCGAAGGTTATGTACAGGAAAGAACTATATTTTTCAAAGATGA
CGGGAACTACAAGACACGTGCTGAAGTCAAGTTTGAAGGTGATACCCTTGTTAATAGAATCGAGTTAA
AAGGTATTGATTTTAAAGAAGATGGAAACATTCTTGGACACAAATTGGAATACAACTATAACTCACACA
ATGTATACATCATGGCAGACAAACAAAAGAATGGAATCAAAGTTAACTTCAAAATTAGACACAACATTG
AAGATGGAAGCGTTCAACTAGCAGACCATTATCAACAAAATACTCCAATTGGCGATGGCCCTGTCCTTT
TACCAGACAACCATTACCTGTCCACACAATCTGCCCTTTCGAAAGATCCCAACGAAAAGAGAGACCACA
TGGTCCTTCTTGAGTTTGTAACAGCTGCTGGGATTACACATGGTATGGATGAACTATACAAAGCAACGC
GTCCGTACGACGTCCCGGACTACGCTGGCTATCCCTATGATGTGCCCGATTATGCGTATCCGTACGATG
TTCCAGATTATGCCGCTGCTAGTTTTTAAATATGCAGAATAAAATAAATAAGTAAATATCATACGTTTAT
ATGTATATATATATATATATATATATATTTGAGACGTATATTTTATATATGCATTATATATACATTCGTATAA
ATATACGTGCATGTAACTTTTTCTATATTATTATTATTTTTTTTATATTTATTATCATTTTTTTTTTTTTCTTA
TGTATTTTTAATATATATATATATATATATATATTTATTTATTTATTTATTAGTAGATAATTTTTTTTATCTTGT
TTTAACTGAAATAATTCTAAATTTTCTTAAATGCAGGAACAAAATATAAACGGTTTATGGTTTTATTTATT
TATATTATATATATGAAAAATATAATTTTTGTTCCATTTACACATAAATGTCACACAAAAGAGAAATAAC
ATAAATGTTTATATTTTTTTTAACCTGCCAATTTTTAGAAGCATAAAATGTTATATTTTTACCAAAAAAAA
AAAAAAAATAAATAAATAAATAAATGAAATATAATAAAAATATAATTAAAATATATAATAATAATAATA
AATAAATGTAAGGCACATTTTTAAAAGACAATATTTATAAGTCCATTTAACATTATTATTTGAGCGTACA
TTACTCCTCTTTTTTATTAAGTGATCGATGCGGCCGCTTAATCATTCTTCTGAGTATTCTATAGTGTCACC
TAAATAGCTTGGCGTAATCATGGTCATAGCTGTTTCCTGTGTGAAATTGTTATCCGCTCACAATTCCACA
CAACATACGAGCCGGAAGCATAAAGTGTAAAGCCTGGGGTGCCTAATGAGTGAGCTAACTCACATTAA
TTGCGTTGCGCTCACTGCCCGCTTTCCAGTCGGGAAACCTGTCGTGCCAGCTGCATTAATGAATCGGCC
AACGCGCGGGGAGAGGCGGTTTGCGTATTGGGCGCTCTTCCGCTTCCTCGCTCACTGACTCGCTGCGCT
CGGTCGTTCGGCTGCGGCGAGCGGTATCAGCTCACTCAAAGGCGGTAATACGGTTATCCACAGAATCA
GGGGATAACGCAGGAAAGAACATGTGAGCAAAAGGCCAGCAAAAGGCCAGGAACCGTAAAAAGGCC
GCGTTGCTGGCGTTTTTCGATAGGCTCCGCCCCCCTGACGAGCATCACAAAAATCGACGCTCAAGTCAG
AGGTGGCGAAACCCGACAGGACTATAAAGATACCAGGCGTTTCCCCCTGGAAGCTCCCTCGTGCGCTC
TCCTGTTCCGACCCTGCCGCTTACCGGATACCTGTCCGCCTTTCTCCCTTCGGGAAGCGTGGCGCTTTCT
CATAGCTCACGCTGTAGGTATCTCAGTTCGGTGTAGGTCGTTCGCTCCAAGCTGGGCTGTGTGCACGAA
CCCCCCGTTCAGCCCGACCGCTGCGCCTTATCCGGTAACTATCGTCTTGAGTCCAACCCGGTAAGACAC
GACTTATCGCCACTGGCAGCAGCCACTGGTAACAGGATTAGCAGAGCGAGGTATGTAGGCGGTGCTAC
AGAGTTCTTGAAGTGGTGGCCTAACTACGGCTACACTAGAAGGACAGTATTTGGTATCTGCGCTCTGCT
GAAGCCAGTTACCTTCGGAAAAAGAGTTGGTAGCTCTTGATCCGGCAAACAAACCACCGCTGGTAGCG
GTGGTTTTTTTGTTTGCAAGCAGCAGATTACGCGCAGAAAAAAAGGATCTCAAGAAGATCCTTTGATCT
TTTCTACGGGGTCTGACGCTCAGTGGAACGAAAACTCACGTTAAGGGATTTTGGTCATGAGATTATCAA
AAAGGATCTTCACCTAGATCCTTTTAAATTAAAAATGAAGTTTTAAATCAATCTAAAGTATATATGAGTA
AACTTGGTCTGACAGTTACCAATGCTTAATCAGTGAGGCACCTATCTCAGCGATCTGTCTATTTCGTTCA

TCCATAGTTGCCTGACTCCCCGTCGTGTAGATAACTACGATACGGGAGGGCTTACCATCTGGCCCCAGT
GCTGCAATGATACCGCGAGACCCACGCTCACCGGCTCCAGATTTATCAGCAATAAACCAGCCAGCCGG
AAGGGCCGAGCGCAGAAGTGGTCCTGCAACTTTATCCGCCTCCATCCAGTCTATTAATTGTTGCCGGGA
AGCTAGAGTAAGTAGTTCGCCAGTTAATAGTTTGCGCAACGTTGTTGGCATTGCTACAGGCATCGTGGT
GTCACGCTCGTCGTTTGGTATGGCTTCATTCAGCTCCGGTTCCCAACGATCAAGGCGAGTTACATGATC
CCCCATGTTGTGCAAAAAAGCGGTTAGCTCCTTCGGTCCTCCGATCGTTGTCAGAAGTAAGTTGGCCGC
AGTGTTATCACTCATGGTTATGGCAGCACTGCATAATTCTCTTACTGTCATGCCATCCGTAAGATGCTTT
TCTGTGACTGGTGAGTACTCAACCAAGTCATTCTGAGAATAGTGTATGCGGCGACCGAGTTGCTCTTGC
CCGGCGTCAATACGGGATAATACCGCGCCACATAGCAGAACTTTAAAAGTGCTCATCATTGGAAAACG
TTCTTCGGGGCGAAAACTCTCAAGGATCTTACCGCTGTTGAGATCCAGTTCGATGTAACCCACTCGTGC
ACCCAACTGATCTTCAGCATCTTTTACTTTCACCAGCGTTTCTGGGTGAGCAAAAACAGGAAGGCAAAA
TGCCGCAAAAAAGGGAATAAGGGCGACACGGAAATGTTGAATACTCATACTCTTCCTTTTTCAATATTA
TTGAAGCATTTATCAGGGTTATTGTCTCATGAGCGGATACATATTTGAATGTATTTAGAAAAATAAACA
AATAGGGGTTCCGCGCACATTTCCCCGAAAAGTGCCACCTGACGTCTAAGAAACCATTATTATCATGAC
ATTAACCTATAAAAATAGGCGTATCACGAGGCCCTTTCGTCTCGCGCGTTTCGGTGATGACGGTGAAAA
CCTCTGACACATGCAGCTCCCGGAGACGGTCACAGCTTGTCTGTAAGCGGATGCCGGGAGCAGACAAG
CCCGTCAGGGCGCGTCAGCGGGTGTTGGCGGGTGTCGGGGCTGGCTTAACTATGCGGCATCAGAGCA
GATTGTACTGAGAGTGCACCATATGCGGTGTGAAATACCGCACAGATGCGTAAGGAGAAAATACCGCA
TCAGGCGCCATTCGCCATTCAGGCTGCGCAACTGTTGGGAAGGGCGATCGGTGCGGGCCTCTTCGCTA
TTACGCCAGCTGGCGAAAGGGGGATGTGCTGCAAGGCGATTAAGTTGGGTAACGCCAGGGTTTTCCCA
GTCACGACGTTGTAAAACGACGGCCAGTGAATTGTAATACGACTCACTATAGAATTCTAGATTTAATAA
ATATGTTCTTATATATAATGAGAAATAAATATTTAACATATGTTTAAAAAGAAAAATTTAAGATTTACAT
GATTAGGAATAAAAATATTAAAAGCTATATATTAATGTTGTACTTTATGTTCATATTGTAAAAGATAAAT
ATAATTTTTATTAAAATTTATATTTATTATTATTATAAGTTCTTAGAAAATACATTTTGTTTTAAAATAATA
CTATAAAAAGAACAATAATAAAAATAAAATACAATTAAAACATAATTAAAAAAGAAAAATACAGACAG
TAAAAAAAATCGCTATCCCATAAATTACAAAACATGAATTAATAAACATATATTGTTTATTAATAAAATT
GTAAGTTATTTTTATATATATACATAATAAATTAAAGATACTGAAACAATAATTTTAAATGTAAATAAATGA
ATAATGATATGTTTTATATGATTCATTATTCTATATTTATAAGGAAGATTACAAAAAAAAAAATTCATATGT
ATTTTTTTTGTAATTTCTGTGTTTATGTTTTTATTTTTATAATATTTTTAATCTATTATTAAATAAGCTTAAT
CATTCTTCTCATATACTTCAAATTTGTACTTAATGCCTTTCTCCTCCTGGACATCAGAGAGAACACCTGGG
TATTCTGGCAGAAGTTTATATTTCTCCAAATCAATTTCTGGAAAAAACGTGTCACTTTCAAAGTCTTGCAT
GATCCTTGTCACAAATAGTTTAAGATGGCCTGGGTGATTCATGGCTTCCTTATAAACAGAACTGCCACC
AACTATCCAGACCATGTCTACTTTATTTGCTAATTCTGGTTGTTCAGTAAGTTTTAAGGCATCATCTAGAC
TTCTGGAAAGAAAATGAGCTCCTTGTGGAGGTTCCTTGAGTTCTCTGCTGAGAACTAAATTAATTCTACC
CTTTAAAGGTCGATTCTTCTCAGGAATGGAGAACCAGGTCTTCTTACCCATAATCACCAGATTCTGTTTA
CCTTCTACTGAAGAGGTTGTGGTCATTCTCTGGAAATATCTAAATTCGTTCCTGAGCGGTGGCCAGGGG
TAGTCCCCGTTCTTGCCGATGCCCATGTTCTGGGACACAGCGACGATGCAGTTTAGCGAACCATGCATG
GATCCTTTTATAAAATTTTTATTTATTTATAAGCAAATATATATTTTTATATATTTATTTTATATTTTGAAAT
ATTTTTAAAATTTAATTTAATTTTTTATTATATACTTTAAAAATGGCAGTTATTAAAAAATAAGGATAAAA
TTATTATTATTTTTAATTGGTGTTTTGTATTTTTTTTTGTTATATAATATTAAATTTTAATTTGGAGAAAAT
GTTTATTGTATTCAATATAATAACATAATATTTATTTCATTTTATTTTAGAAATACACGTATTTTTTTTTCA
ACTCTATACTATGTACTTATGTGTATAAATTATTAAAATAAATAAAAATATTATAAAATGCATATAATGTA
GGGATAAAATATTACTATATTTATAGTGTCAGTGCAGGTGTAAAATCCTCGGAGTATAAAAAAATAAAA
TGGATTATTTAAAAAAAGGTACTGCATAAAACATTTAATTTGATGCTTGTAGATGAGTTAAG

Plasmid features:

EF1-alpha elongation fator 1 alpha of *Plasmodium falciparum*

XhoI enzyme cleavage site

PfA-M1 + GFP (*Plasmodium falciparum* aminopeptidase coding sequence without signal peptide and stop codon, in frame with GFP)

Guanine added to keep PfA-M1 in frame with GFP and hemagglutinin

PstI enzyme cleavage site

3x hemagglutinin coding sequence

CAM 3´UTR (3´untranslated region from *Plasmodium falciparum* calmodulin gene)

Beta lactamase coding sequence (ampicillin resistance)

HRP 3´ UTR (3´untranslated region from the histidine rich protein of *Plasmodium falciparum*)

hDHFR (human dihydrofolate reductase coding sequence, resistance to WR99210)

EF-1 beta (elongation fator 1 beta promoter from *Plasmodium falciparum*)
